# Supplementary material for: Kinesiophobia and its correlations with pain, proprioception, and functional performance among individuals with chronic neck pain
Source: PLoS One. 2021 Jul 8;16(7):e0254262. doi: 10.1371/journal.pone.0254262 (PMC8266083; doi:10.1371/journal.pone.0254262)
Supplement: S1 Data — (DOCX) [file pone.0254262.s001.docx]

**STUDY PROTOCOL**

In the general population, chronic neck pain (CNP) is one of the debilitating conditions that can impair the ability to perform regular everyday activities, decrease productivity, and adversely affect life quality. In developed countries, approximately two-thirds of people experience neck pain. At a given point in time, about 14% to 16% of the adult population globally experience neck pain, and the mean lifetime prevalence is 48.5%.

Chronic pain is categorized as pain that lasts more than three months. However, there are distinct pathological mechanisms that contribute to the development of chronic musculoskeletal pain. It is critical to understand neuroplasticity (a neuron's capacity to completely alter its structure, function, or biochemical profile in response to repeated afferent sensory inputs) to know how acute pain transforms as chronic pain. Local inflammation of the injured tissue increases peripheral sensory neurons' sensitivity (nociceptors), resulting in repetitive abnormal afferent input to the central nervous system. Researchers also discovered that people with chronic pain have less volume in their prefrontal cortex—the part of the brain that controls thoughts, personality expression, and social behavior. Chronic pain has been shown to induce escape and avoidance behaviors and is strongly associated with kinesiophobia.

Kinesiophobia is a concept that describes a condition in which a patient has an unwarranted and deteriorating fear of physical movement and actions that results from a feeling of vulnerability to painful injury or re-injury. An exaggerated negative cognitive and affective response to an anticipated or actual pain is expressed as pain catastrophizing. It is characterized by an increase in the possible negative aspects of pain, an inability to disengage from stressful thinking, and a sense of helplessness in dealing with pain. In the acute pain stage, these habits may be adaptive. However, in long-lasting pain, the issue paradoxically worsens, aggravating impairment and pain perception thresholds as patients enter a vicious cycle that perpetuates chronic pain and functional disability.

CNP is multifactorial, and the factors that contribute to maintain and increase pain intensity are hard to define. A closed-loop, proprioceptive, vestibular, and visual systems' interplay role works to maintain static and dynamic balance during functional tasks. Kinesiophobia and catastrophic behaviors can induce neck pain recurrence or cause changes in the somatosensory system. The cervical afferent input to the higher centers may be changed by these changes, thereby impairing the cervical proprioception, which warrants assessment in detail. Previous studies have shown that CNP individuals may be difficult or incapable of performing functional tasks. Also, kinesiophobia can further hinder their overall functional performance, which can affect their quality of life. Due to the avoidance of physical exercise, kinesiophobia may contribute to a deterioration of functional ability, leading to decreased mobility and chronic pain. However, there is a controversy in the literature and there is no conclusive evidence of the impact of kinesiophobia on functional performance among individuals with CNP.

AIMS AND OBJECTIVES

It is vital to develop effective recovery strategies in these neck pain patients by evaluating psychological factors before and after rehabilitation and recognizing which psychological impairments contribute significantly to neck rehabilitation. There is no explored research, to our knowledge, in neck pain patients that correlated pain, proprioception and functional performance with kinesiophobia. The objectives of this study are 1) to evaluate the relationship between kinesiophobia, neck pain intensity, proprioception, and functional performance; 2) to determine whether kinesiophobia predicts pain intensity, proprioception, and functional performance among CNP individuals. We hypothesize that kinesiophobia is significantly associated with pain, proprioception, and functional performance. This study's results may provide a fundamental understanding of the interactions between kinesiophobia, pain, proprioception, functional performance, and clinical management characteristics of kinesiophobia in CNP patients.

### Study Population:

**Inclusion criteria**:

- Neck pain for more than three months
- Chronic neck pain elicited by neck postures, neck movements, or palpation of the cervical musculature.
- Neck pain intensity of 30 to 70 mm measured on a visual analog scale (VAS); and
- Neck disability score, 15 or more measured on neck disability index (NDI).

**Exclusion criteria**

- Signs of radiculopathy that were tested and confirmed by a positive Upper Limb Tissue Stress Test and Spurling Test
- History of neurological disease or whiplash injury
- Cervical myelopathy
- Tumors
- Cervical spine infection
- Insufficiency of the vertebrobasilar artery.

Procedure

Following the standard protocol, anthropometric characteristics were measured, including height (m), weight (kg), and BMI (kg/m2).

**Kinesiophobia assessment**

The Tampa Scale of Kinesiophobia (TSK) assessed the fear of movement or re-injury. There was an appropriate degree of internal consistency in this questionnaire's original versions (Cronbach's alpha of 0.8), proof of prejudice, and parallel criterion-related and gradual validity. The total TSK score ranges from 17 to 68, where 17 indicates no kinesiophobia, 68 shows moderate kinesiophobia, and ± 37 indicates that kinesiophobia is present.

**Pain Assessment**

The current neck pain intensity was assessed using the visual analog scale (VAS) scores. The scale is 100 mm long and is anchored by the words "no pain" and "worst pain imaginable "on the left and right sides. Individuals were requested to draw a vertical mark that better reflects the pain level across the horizontal line; 0- 30 mm indicates mild, 30 – 70 mm indicates moderate, and > 70 mm indicates severe pain intensity. VAS is a widely used evaluation method and has good reliability and validity.

**Proprioception testing**

The Cervical Range of Motion (CROM) device (Performance Attainment Associates, Minnesota) was used to assess cervical spine range of motion (ROM) and proprioception. The CROM unit has three inclinometers and a magnetic yoke or harness. The JPE is estimated according to the participant’s capacity to consciously reposition his or her head to a target location previously shown by the examiner. Everyone was guided to the testing lab and got familiarized with the testing procedures. The individuals were asked to sit in the chair and put on the CROM device as if it were a pair of glasses, which was then secured around the head using the Velcro band. The individual in this manuscript has given written informed consent to publish these case details. A magnetic yoke was positioned directly over the participant's shoulders and pointed north. The examiner used a webbing strap to minimize the patients' shoulder and trunk motions during the examination. The examiner asked the individual to maintain the head in the neutral position (starting point) and standardized the CROM device to the starting position.

To start with JPE testing, the participants were asked to close their eyes throughout the testing procedure. The examiner slowly guided the participant head to the target position, which was previously determined and is 50% of their maximum ROM. The participant head was then held in the target position for three seconds, allowing the individuals to memorize the target position. Successively, the participant head was brought back to a starting position. The participant was then asked to reposition their heads to the target position consciously (absolute error). The examiner measured the relocation accuracy in degrees once they reached the reference position. The JPE testing was performed in four directions, i.e., flexion, extension, left rotation, and right rotation. A simple chit method was used to randomize the order of testing four directions of JPE testing. Three attempts were performed in each movement direction, and an average of three attempts was used for analysis.

**Functional Performance**

Handgrip strength as a measure of functional performance was measured using the Baseline® hydraulic hand dynamometer. It is a valid, clinically easy, useful test to measure grip strength and identify any functional performance changes in individuals with CNP. This test is performed with the participant sitting in the chair with the shoulder adducted and neutrally rotated, elbow flexed to 90 degrees, forearm and wrist maintained in the neutral position (neither flexed nor extended) and gripping the handheld dynamometer. The individual in this manuscript has given written informed consent to publish these case details. The dynamometer was set to the second or third handle position to ensure consistency, claimed to be more suitable by the participant. For most participants, the second position was used, which was considered the optimal level for grip evaluation and was adopted for routine testing by the American Society of Hand Therapists. The individuals squeezed the handheld dynamometer's handle as hard as possible, as explained by the investigator. The measurements were performed on the dominant side, three trials were conducted, and an average of three trials were used for analysis. The handgrip strength was recorded in kilograms. Between each attempt, a one-minute rest period could minimize fatigue effects. No verbal encouragement was to the participants during the handgrip strength measurements. The hydraulic hand dynamometer was calibrated regularly throughout the study duration.
