# Supplementary material for: Kinesiophobia and its correlations with pain, proprioception, and functional performance among individuals with chronic neck pain
Source: PLoS One. 2021 Jul 8;16(7):e0254262. doi: 10.1371/journal.pone.0254262 (PMC8266083; doi:10.1371/journal.pone.0254262)
Supplement: S1 File — (PDF) [file pone.0254262.s003.pdf]

Tampa Scale for Kinesiophobia

1 = strongly disagree
2 = disagree
3 = agree
4 = strongly agree

1. I'm afraid that I might injury myself if I exercise	1	2	3	4
2. If I were to try to overcome it, my pain would increase	1	2	3	4
3. My body is telling me I have something dangerously wrong	1	2	3	4
4. My pain would probably be relieved if I were to exercise	1	2	3	4
5. People aren't taking my medical condition seriously enough	1	2	3	4
6. My accident has put my body at risk for the rest of my life	1	2	3	4
7. Pain always means I have injured my body	1	2	3	4
8. Just because something aggravates my pain does not mean it is dangerous	1	2	3	4
9. I am afraid that I might injure myself accidentally	1	2	3	4
10. Simply being careful that I do not make any unnecessary movements is the safest thing I can do to prevent my pain from worsening	1	2	3	4
11. I wouldn't have this much pain if there weren't something potentially dangerous going on in my body	1	2	3	4
12. Although my condition is painful, I would be better off if I were physically active	1	2	3	4
13. Pain lets me know when to stop exercising so that I don't injure myself	1	2	3	4
14. It's really not safe for a person with a condition like mine to be physically active	1	2	3	4
15. I can't do all the things normal people do because it's too easy for me to get injured	1	2	3	4
16. Even though something is causing me a lot of pain, I don't think it's actually dangerous	1	2	3	4
17. No one should have to exercise when he/she is in pain	1	2	3	4

Scoring Information
Tampa Scale for Kinesiophobia

A total score is calculated after inversion of the individual scores of items 4, 8, 12 and 16.
