# Supplementary material for: Kinesiophobia and its correlations with pain, proprioception, and functional performance among individuals with chronic neck pain
Source: PLoS One. 2021 Jul 8;16(7):e0254262. doi: 10.1371/journal.pone.0254262 (PMC8266083; doi:10.1371/journal.pone.0254262)
Supplement: Attachment — Submitted filename: Response to Reviewers.doc [file pone.0254262.s005.doc]

Thank you for your effort and time in reviewing our manuscript. The reviewing process has significantly improved the quality of this manuscript. I am submitting this "Response to reviewers" document summarizing the changes that we made in response to the critiques.

| **RESPONSE TO REVIEWR 1** | | |
| --- | --- | --- |
| 1. 1 | You have mainly addressed the reviewers' concerns however However simply say that only absolute errors were recorded as a limitation is not acceptable. Constant and Variable errors can be calculated from the data that you have. There are many articles with the formulas. Here is one of many that explains how to calculate absolute, constant and variable error. https://motorcontrol.wordpress.com/2008/06/19/constant-error-variable-error-absolute-error-and-root-mean-square-error-labview/#:~:text=The%20formula%20for%20it%20is,alone%20without%20mentioning%20the%20direction. | - Thank you for sharing the link which showed the formulas to calculate the constant and variable error. - We are unfortunate that we could not calculate the Constant and variable errors. - The formula to measure constant error is: Σ (xi-T)/N, and variable error is sq. root (Σ (xi-M)^2/N. In the formula "Xi" indicates the deviation from the target; it comes with a positive (overshooting) or negative sign (undershooting) that points out the error's direction. We did not measure or record this overshooting or undershooting. We measured the total error around the target (absolute error) only. |
